# Spinal cord injury – assessing tolerability and use of combined rehabilitation and NeuroAiD (SATURN) study – primary results of an exploratory study

**DOI:** 10.1080/10790268.2022.2067972

**Published:** 2022-05-23

**Authors:** Ramesh Kumar, Ohnmar Htwe, Azmi Baharudin, Shaharuddin Abdul Rhani, Kamalnizat Ibrahim, Jagdeep Singh Nanra, Muhindra Gsangaya, Hezery Harun, Khairrudin Kandar, Maatharasi Balan, Shawn Peh, Yogesh Pokharkar, Abhinay Ingole, Mohammad Hisam Ariffin

**Affiliations:** 1 Neurosurgery Unit, Department of Surgery, Faculty of Medicine, Universiti Kebangsaan Malaysia Medical Centre, Kuala Lumpur, Malaysia; 2 Department of Orthopaedics and Traumatology, Faculty of Medicine, Universiti Kebangsaan Malaysia Medical Centre, Kuala Lumpur, Malaysia; 3 Department of Orthopaedic, KPJ Selangor Specialist Hospital, Selangor, Malaysia; 4 Department of Orthopaedic, KPJ Ampang Puteri Specialist Hospital, Selangor, Malaysia; 5 Prince Court Medical Centre, Kuala Lumpur, Malaysia; 6 Department of Orthopaedics and Traumatology, Hospital Serdang, Selangor, Malaysia; 7 Department of Orthopaedics and Traumatology, Hospital Pengajar Universiti Putra Malaysia, Selangor, Malaysia; 8 Department of Orthopaedic, Avisena Specialist Hospital, Selangor, Malaysia; 9 Singapore Clinical Research Institute, Singapore, Singapore

**Keywords:** MLC601, MLC901, Spinal cord injury, SATURN study, American Spinal Injury Association Impairment Scale, Recovery

## Abstract

**Objective:**

MLC601/MLC901 has demonstrated neuroprotective and neuroregenerative properties that enhance neurological recovery in stroke and traumatic brain injury. We aimed to evaluate its safety and potential efficacy in patients with severe spinal cord injury.

**Methods:**

Patients with American Spinal Injury Association (ASIA) Impairment Scale (AIS) A and B were included in an open-label cohort study. Each received a course of MLC601/MLC901 for 6 months in addition to standard care and rehabilitation. Key endpoints were safety, AIS grade and motor scores at month 6 (M6).

**Results:**

Among 30 patients included (mean age 42.2 ± 17.6 years, 24 men), 20 patients had AIS A while 10 patients had AIS B at baseline. Ten patients experienced 14 adverse events including one serious adverse event and six deaths, none were considered treatment-related. AIS improved in 25% of AIS A and 50% of AIS B. Improvement in ASIA motor score was seen most with cervical injury (median change from baseline 26.5, IQR: 6–55). These findings appear to be better than reported rates of spontaneous recovery for SCI AIS A and B.

**Conclusion:**

MLC601/MLC901 is safe and may have a role in the treatment of patients with SCI. A controlled trial is justified.

## Introduction

Spinal cord injury (SCI) leads to devastating consequences of severe neurological and functional deficits. It impacts the quality of life for survivors and their families with the lifetime cost of care ranging from $1.2 to more than $5.1 million per patient.^1,2^ SCI produces a cascade of deleterious processes which causes an accumulation of glutamate in the extracellular compartment and excitotoxicity of neighboring neurons, processes on which MLC601/MLC901 have shown their neuroprotective effect. There are currently no proven pharmacological therapies to augment motor function and functional recovery in SCI. Few pharmacologic therapies for SCI, among which are methylprednisolone, which have shown limited benefit/risk ratio with modest efficacy and possible severe complications,^3^ and monosialic ganglioside GM1 which did not show any significant sustained efficacy and possible severe complications.^4^

In this exploratory study, we evaluated the safety of MLC601/MLC901 and their potential to augment functional and motor recovery after severe SCI.

## Methods

The full study protocol was approved by the ethics committee of the Hospital University Kebangsaan Malaysia and has been published.^5^ All patients included in the study provided informed consent.

Briefly, SATURN (ClinicalTrials.gov NCT02537899) was designed to evaluate the safety and potential role of MLC601/MLC901 in addition to rehabilitation and standard of care in SCI. Inclusion criteria were age of 18–65 years, diagnosis of SCI between 3 days and 4 weeks at the time of study entry, categorized as American Spinal Injury Association (ASIA) Impairment Scale (AIS) A or B.^6^ Patients with non-survivable injury or with serious comorbidities and other conditions that would limit clinical assessment were excluded. Patients received a 6-month course of either MLC601 (4 capsules) or MLC901 (2 capsules) 3 times a day orally in addition to rehabilitation and standard of care. Patients were assessed at months (M) 1, 3 and 6.

The key safety endpoint was the occurrence of adverse events (AEs) during the treatment course. Key efficacy endpoints were improvements in the AIS grade and the ASIA total motor score at M6 compared to baseline. The improvement in AIS grade was analyzed by binomial test, to test the hypotheses of proportion equal to zero and improvement in the ASIA total motor score at M6 was analyzed by Wilcoxon signed-rank test to test the hypotheses of median equal to zero. Missing AIS grade or ASIA motor scores were imputed by the last observation carried forward except for patients who died prior to 6 months.

## Results

**Figure 1:**
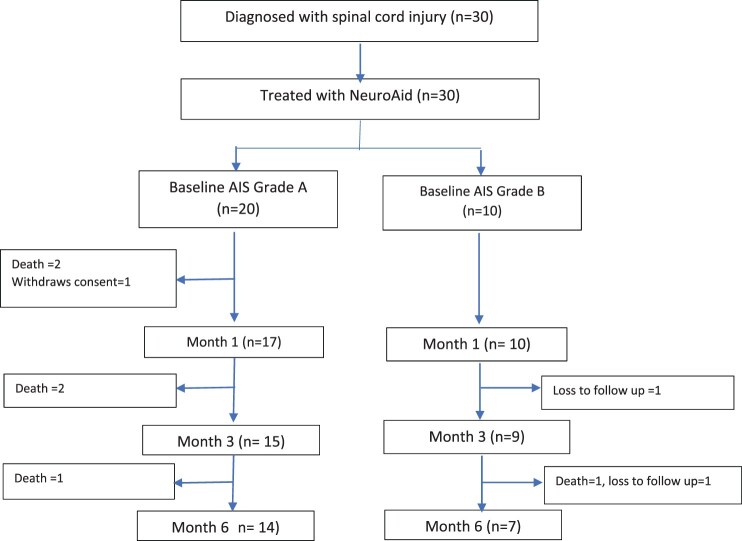
Consort diagram of patient flow.

A total of 30 patients were included, of which 20 were AIS A and 10 were AIS B at baseline (Figure 1). The demographic profile and baseline characteristics grouped by AIS A and B are presented in Table 1.

**Table 1:** Demographics and baseline characteristics.

Characteristic	AIS grade A (*N* = 20)	AIS grade B (*N* = 10)
**Age**	** **	
Median (IQR)	38.2 (27.1)	36.1 (20.0)
Minimum, Maximum	20.9, 72.2	17.6, 73.9
**Sex**	** **	
Male	18 (90.0)	6 (60.0)
Female	2 (10.0)	4 (40.0)
**Race/Ethnicity**	** **	
Chinese	6 (30.0)	3 (30.0)
Indian	0	0
Malay	11 (55.0)	6 (60.0)
Other	3 (15.0)	0
**Time from SCI to baseline assessment** (**days)**		
Median (IQR)	15.5 (17.0)	14.0 (24.0)
Minimum, Maximum	4, 36	2, 105
**Presence of other Injury**	** **	
Chest	1 (5.0)	0
Abdominal	2 (10.0)	0
Pelvic	1 (5.0)	1 (10.0)
Limb (Upper and lower limb)	3 (15.0)	0
Others	3 (15.0)	0
**Imaging**	** **	
Magnetic Resonance Imaging	11 (55.0)	9 (90.0)
Computed Tomography	9 (45.0)	1(10.0)
**Level of injury**	** **	
Cervical	9 (45.0)	4 (40.0)
Thoracic	9 (45.0)	6 (60.0)
Lumbar	2 (10.0)	0
**Cause of a SCI**	** **	
Motor vehicular accident	8 (40.0)	5 (50.0)
Fall	9 (45.0)	1 (10.0)
Post-operative	0	2 (20.0)
Tuberculosis	1 (15.0)	1 (10.0)
Other	2 (10.0)	1 (10.0)
**Other medical conditions and comorbidities**		
Respiratory failure	2 (10.0)	2 (20.0)
Spasticity	4 (20.0)	2 (20.0)
Bladder retention	15 (75.0)	3 (30.0)
Bowel retention	15 (75.0)	3 (30.0)
Sexual dysfunction	8 (40.0)	5 (50.0)
Pneumonia	6 (30.0)	0
Autonomic dysreflexia	1 (5.0)	0
Pain	0	1 (10.0)
Bladder incontinence	8 (40.0)	6 (60.0)
Bowel Incontinence	7 (35.0)	6 (60.0)
Others	1 (5.0)	1 (10.0)
**Surgical intervention perform**	** **	
Yes	18 (90.0)	9 (90.0)
No	2 (10.0)	1 (10.0)
**Rehabilitation provided**	** **	
Physical	19 (95.0)	6 (60.0)
Occupational	15 (75.0)	6 (60.0)

AIS, American Spinal Injury Association Impairment Scale; SD, standard deviation; IQR, interquartile range; SCI, spinal cord injury.

During the course of the study, 10 patients experienced 14 AEs. Six patients (baseline AIS grade A *n* = 5 (all cervical), grade B *n* = 1) died due to SCI-related complications, i.e. septicemia due to urinary tract infection, infected sacral sore and severe pneumonia. Other AEs reported were hyponatremia, hypoglycemia, pneumonia, neuropathic pain, fever, urinary tract infection and diaphoresis which were reported in 4 patients (13.3%). None were deemed definitely, likely or possibly related to MLC601/MLC901 based on the WHO-UMC system for standardized case causality assessment.

**Figure 2:**
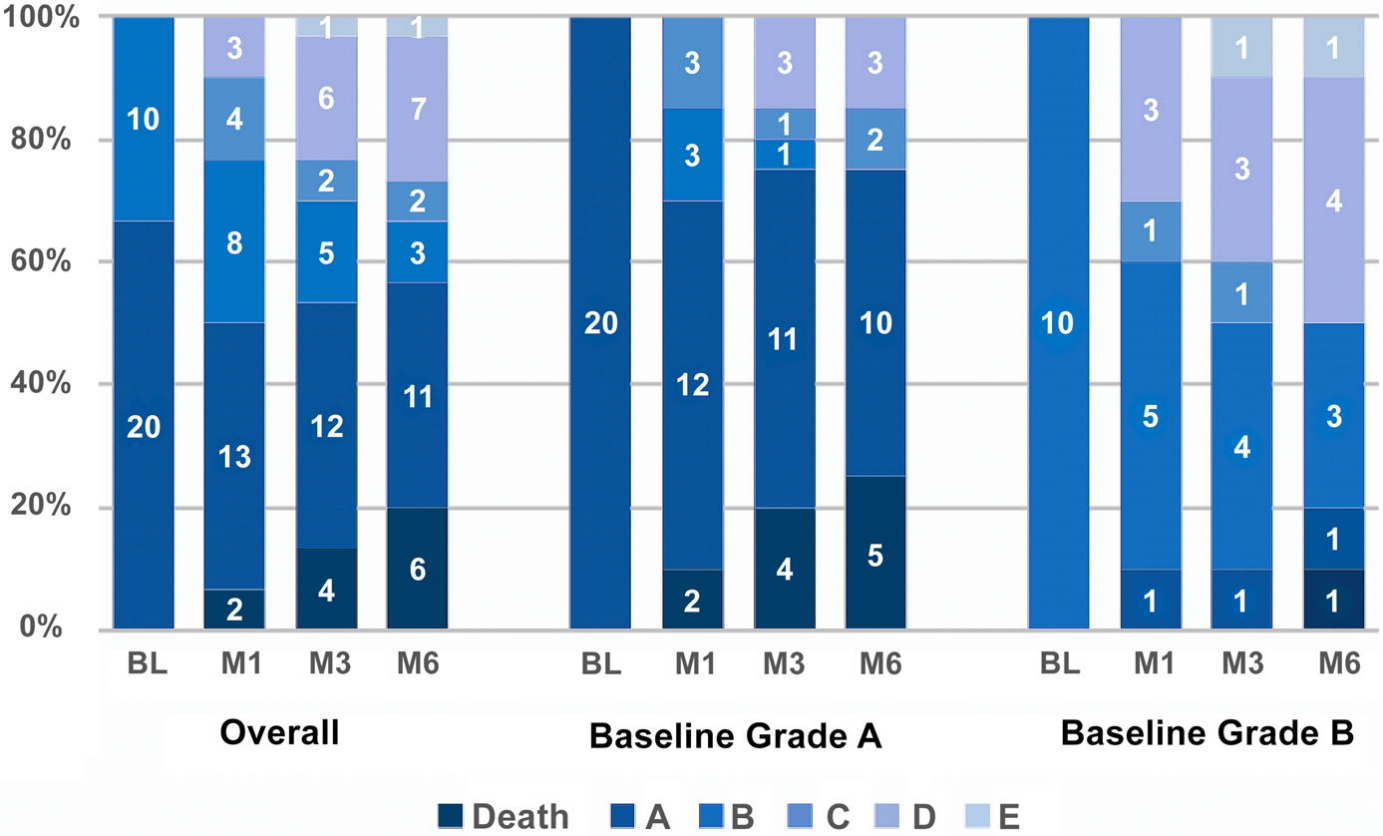
AIS grading at baseline and months 1, 3 and 6 of SATURN patients (*n* = 30). Numbers on the bars refer to number of patients with AIS grade.

Overall, 33% of patients improved on AIS grading at month 6 compared to baseline: 5 (25%) among the 20 patients with AIS A and 5 (50%) among the 10 patients with AIS B. A most robust conversion was exhibited by AIS B patients. At M1 and M3, 40% and 50% of AIS B patients improved in AIS grading, compared to 30% and 25% among AIS A patients, respectively (Figure 2).

The Asia total motor score improved over time from 17.1 at baseline to 49.9 at M6 with a median change from baseline at 26.5 (IQR 6–55; *P* < 0.05). The improvement was seen best in the group with cervical injury (Table 2).

**Table 2:** Median total motor scores at baseline and months 1, 3, and 6 of SATURN patients overall and by level of injury.

Group/Level of injury	Timepoint	*n*	Total motor score		
			Median (IQR)	Median change from baseline (IQR)	P value*
**Overall**	Baseline	30	50 (20–50)		
	Month 1	28	50 (28.5–50)	0 (0–8.5)	0.001
	Month 3	26	50 (50–70)	0.5 (0–31)	<0.001
	Month 6	24	54 (50–81)	9 (0–43.5)	0.001
**Cervical**	Baseline	13	20 (5–20)		
	Month 1	11	27 (0–34)	2 (0–9)	0.023
	Month 3	9	35 (3–60)	6 (1–40)	0.023
	Month 6	8	48.5 (21–81)	26.5 (6–55)	0.016
**Thoracic**	Baseline	15	50 (50–50)		
	Month 1	15	50 (50–50)	0 (0–6)	0.125
	Month 3	15	50 (50–87)	0 (0–31)	0.063
	Month 6	14	54 (50–92)	4 (0–28)	0.039
**Lumbar**	Baseline	2	55 (50–60)		
	Month 1	2	60 (50–70)	5 (0–10)	Not done
	Month 3	2	60 (50–70)	5 (0–10)	Not done
	Month 6	2	59 (50–68)	4 (0–8)	Not done

*By Wilcoxon signed-rank test.

## Discussion

In this open-label exploratory study, we found MLC601/MLC901 to be safe as an add-on treatment in severe SCI. None of the AEs were considered related to MLC601/MLC901. This safety profile is similar to that observed in previous studies on MLC601/MLC901.^7,8^ The proportion of death was not unexpected for a cohort of patients with severe SCI, especially in cervical grade A SCI. A third of our cohort of patients with severe SCI treated with MLC601/MLC901 improved on AIS grading with corresponding motor recovery by M6, one patient even achieved complete recovery (grade E).

MLC601/MLC901 was shown to have neuroprotective and neuroregenerative properties in preclinical models and clinical trials of traumatic and ischemic brain injuries. Studies in both in vitro and in vivo experiments showed a strong protective effect against glutamate-induced cell death, increase in BDNF expression, enhanced neurogenesis, promotion of cell proliferation, stimulation of neurite growth and effects on neuroinflammation.^9–12^

Our observation of 25% improvement in patients with AIS A and 50% in patients with AIS B and corresponding motor recovery over the 6-month period while on study treatment appears to be higher than previously reported rates of spontaneous recovery and clinical trials. Previously reported spontaneous recovery rates in patients with AIS A were reportedly 10% for conversion to AIS B (i.e. some sensory function) and about 10% for regaining some motor function (i.e. AIS C) while 80% of the initial AIS A patients remain as AIS A. AIS B conversion to AIS C was between 15% and 40%, and AIS B conversion to AIS D was as much as 40%.^13^ Likewise, the improvement in our SATURN cohort is comparable or better than those seen in other open-label therapeutic exploratory studies on severe SCI that reported improvement rates of 21.2% to 26.3%.^14–16^ (Supplemental Table A, B, C).

We may consider any improvement in AIS grading as clinically important. For planning a controlled clinical trial with improvement in AIS grade at 6 months as a primary endpoint, a sample size of 786 (393 per treatment arm) would be required to achieve 80% power to detect a difference of 9% (i.e. 33% in Saturn vs. 24% in Control) at 5% significance level. This may be, preceded by a pilot phase to determine the feasibility of the main study with 216 patients with 80% power and α 0.5.

## Conclusions

MLC601/MLC901 is safe in patients with severe SCI. The degree of improvements observed in AIS grade and motor score over time among our treated patients suggest a potential role of MLC601/MLC901 in severe SCI. These findings are encouraging for planning future controlled clinical trials.

## Disclaimer statements

**Contributors** None.

**Conflicts of interest** Authors have no conflict of interests.

## Supplementary Material

Supplemental Material
