# Supplementary material for: Spinal cord injury – assessing tolerability and use of combined rehabilitation and NeuroAiD (SATURN) study – primary results of an exploratory study
Source: J Spinal Cord Med. 2022 May 23;46(4):682–6. doi: 10.1080/10790268.2022.2067972 (PMC10274548; doi:10.1080/10790268.2022.2067972)
Supplement: Supplemental Material [file YSCM_A_2067972_SM6554.docx]

Supplement

Table A. Comparison of neurological and functional outcomes of SATURN study and historical cohorts.

A. Study and participant data

|  | Saturn (N=30) | Derakhshanrad et al (N=19) | Saberi et al (N=52) |
| --- | --- | --- | --- |
| Age in years (years) | 42.2 (17.6) | 29.0 (18–44) | 29.3 (0.9) |
| Gender, Male | 24 (80.00) | 13 (68.5) | 39 (75.0) |
| Cause of SCI |  |  |  |
| MVA | 13 (43.33) | 13 (68.5) | 38 (73.1) |
| Fall | 10 (33.33) | 2 (10.5) | 6 (11.5) |
| Other | 3 (10) | 4 (21.0) | 8 (15.3) |
| Time from SCI to baseline (days), Mean (SD) | 22.2 (24.4) | 245 (14-602) | 280.7 (32.9) |

n (%): number and percentage of patients based on N, SCI: Spinal Cord Injury, MVA: Motor

Table B. Improvement of the impairment grades of patients based on the American Spinal Injury Association grading system during observational period.

|  | Saturn (N=30) | Derakhshanrad et al (N=19) | Saturn vs.Derakhshanrad et al | | Saberi et al (N=52) | Saturn vs. Saberi et al | |
| --- | --- | --- | --- | --- | --- | --- | --- |
|  | N (%) | N (%) | Difference in % (95% CI) | P-value | N (%) | Difference in % (95% CI) | P-value$ |
| AIS grade |  |  |  |  |  |  |  |
| A | 20 (66.7) | 12 (63.2) |  |  | 31 (59.6) |  |  |
| B | 10 (33.3) | 7 (36.8) |  |  | 21 (40.4) |  |  |
|  |  |  |  |  |  |  |  |
| AIS Grade Conversion from Baseline to 6mos |  |  |  |  |  |  |  |
| Patients achieving better grade compared  to baseline | 10/30 (33.3) | 5/19 (26.3) | 7.0 (-21.3, 32.2) | 0.754 | 11/52 (21.2) | 12.2 (-7.89, 33.3) | 0.295 |
| Patients with Baseline Grade A achieving  Grade B or Better Compared to Baseline | 5/20 (25.0) | 5/12 (41.7) | -17.7 (-50.5,17.3) | 0.438 | 10/31 (32.3) | -7.3 (-31.7, 20.0) | 0.755 |
| Patients with Baseline Grade B achieving  Grade C or Better Compared to Baseline | 5/10 (50.0) | 0/7 (0) | 50.0 (30.8,81.6) | 0.044 | 1/21 (4.8) | 45.2 (10.3, 76.3) | 0.008 |

Table. C. Changes in American Spinal Injury Association International Standard for Neurological Classification of Spinal Cord Injury Total Motor Score

| Parameter | Saturn (N=30) | Derakhshanrad et al (N=19) | Saturn (N=30) | Yang et al (N=102) | Saturn vs. Yang et al | |
| --- | --- | --- | --- | --- | --- | --- |
| Visit | Median (IQR) | Median (IQR) | Mean (SD) | Mean (SD) | Mean difference (95% CI)* | P-value* |
| Motor score |  |  |  |  |  |  |
| Baseline | 50.0 (20.0-50.0) | 36.0 (18.0–50.0) | 35.7 (20.0) | 44.44 (20.87) | -8.74 (-18.6, 1.1) | 0.081 |
| Month 6 | 54.0 (50.0-81.0) | 46.0 (22.0–50.0) | 59.5 (26.7) | 46.73 (21.20) | 12.8 (0.8, 24.8) | 0.034 |

References:

1. Kumar R, Htwe O, Baharudin,Ariffin MH, Abdul Rhani S, Ibrahim K, Rustam , Gan R. Spinal Cord Injury-Assessing Tolerability and Use of Combined Rehabilitation and NeuroAiD (SATURN Study): Protocol of An Ex-ploratory Study in Assessing the Safety and Efficacy of NeuroAiD Amongst People Who Sustain Severe Spinal Cord Injury. JMIR Res Protoc. 2016; 5(4): e230.
2. Derakhshanrad N, Saberi , Yekaninejad MS, Eskandari G, Mardani A, Rahdari F, Meybodi KT. Safety of granulocyte colony-stimulating factor (G-CSF) administration for postrehabilitated motor complete spinal cord injury patients: an open-label, phase I study. Cell Transplant. 2013;22 Suppl 1:S139-46.
3. Saberi H, Derakhshanrad N, Yekaninejad MS .Comparison of neurological and functional outcomes after administration of granulocyte-colony-stimulating factor in motor-complete versus motor-incomplete postrehabilitated, chronic spinal cord injuries: a phase I/II study. Cell Transplant. 2014; 23 Suppl 1:S19-23.
4. Fehlings MG, Chen Y, Aarabi B, Ahmad F, Anderson K.D, Dumont T, Fourney DR, Harrop JS, Kim KD, Kwon BK, Lingam HK, Rizzo M, Shih LC, Tsai EC, Vaccaro A, McKerracher L. (2021). A Randomized Controlled Trial of Local Delivery of a Rho Inhibitor (VX-210) in Patients with Acute Traumatic Cervical Spinal Cord Injury. J Neurotrauma. doi: 10.1089/neu.2020.7096.
5. Yang Y, Pang M, Du C,Liu ZY, Chen ZH,Wang NX, Zhang LM, Chen YY,Mo J,Dong JW, Xie PG, Wang QY,Liu B, Rong L .Repeated subarachnoid administrations of allogeneic human umbilical cord mesenchymal stem cells for spinal cord injury: a phase 1/2 pilot study. Cytotherapy. 2021 ;23(1):57-64.
